# Role of ADAM17 in invasion and migration of CD133-expressing liver cancer stem cells after irradiation

**DOI:** 10.18632/oncotarget.8112

**Published:** 2016-03-16

**Authors:** Sung Woo Hong, Wonhee Hur, Jung Eun Choi, Jung-Hee Kim, Daehee Hwang, Seung Kew Yoon

**Affiliations:** ^1^ The Catholic University Liver Research Center and WHO Collaborating Center of Viral Hepatitis, College of Medicine, The Catholic University of Korea, Seoul, Republic of Korea; ^2^ Department of New Biology and Center for Plant Aging Research, Institute for Basic Science, DGIST, Daegu, Republic of Korea

**Keywords:** cancer stem cells, hepatocellular carcinoma, radioresistance, ADAM17, migration

## Abstract

We investigated the biological role of CD133-expressing liver cancer stem cells (CSCs) enriched after irradiation of Huh7 cells in cell invasion and migration. We also explored whether a disintegrin and metalloproteinase-17 (ADAM17) influences the metastatic potential of CSC-enriched hepatocellular carcinoma (HCC) cells after irradiation. A CD133-expressing Huh7 cell subpopulation showed greater resistance to sublethal irradiation and specifically enhanced cell invasion and migration capabilities. We also demonstrated that the radiation-induced MMP-2 and MMP-9 enzyme activities as well as the secretion of vascular endothelial growth factor were increased more predominantly in Huh7^CD133+^ cell subpopulations than Huh7^CD133−^ cell subpopulations. Furthermore, we showed that silencing ADAM17 significantly inhibited the migration and invasiveness of enriched Huh7^CD133+^ cells after irradiation; moreover, Notch signaling was significantly reduced in irradiated CD133-expressing liver CSCs following stable knockdown of the ADAM17 gene. In conclusion, our findings indicate that CD133-expressing liver CSCs have considerable metastatic capabilities after irradiation of HCC cells, and their metastatic capabilities might be maintained by ADAM17. Therefore, suppression of ADAM17 shows promise for improving the efficiency of current radiotherapies and reducing the metastatic potential of liver CSCs during HCC treatment.

## INTRODUCTION

Hepatocellular carcinoma (HCC) is one of the most devastating malignancies worldwide and is highly prevalent in sub-Saharan Africa and Southeast Asia; moreover, its incidence is currently increasing in Western countries [1, 2]. Although remarkable advances in the treatment of HCC have been made, its prognosis is poor due to a high tumor recurrence rate that exceeds 70% after surgical resection [3] and progressive liver failure caused by underlying liver cirrhosis. Among multiple modalities for the treatment of HCC, radiotherapy has been applied in selected cases. Because liver tissue is highly sensitive to radiation, high-dose conventional radiotherapy against HCC is generally limited due to radiation-induced liver injury. To compensate for this drawback, stereotactic body radiation therapy (SBRT) is recommended for HCC; however, evidence of beneficial survival outcomes for advanced HCC remains insufficient [4]. Recently, the hazards of radiotherapy have been shown in cancer biology and clinically based studies, such as enhancement of cancer cell invasiveness *in vitro* [5] and an increase in distant metastasis in some cancer patients [6, 7]. However, the mechanisms underlying metastasis in HCC after irradiation have not been clarified.

Growing evidence reveals that a subpopulation of tumor cells harboring the ability to propagate, called cancer stem cells (CSCs) or cancer stem-like cells (CSLCs), is responsible for tumor initiation, progression and metastasis. In addition, recent studies have described that CSCs in a variety of human tumors play a key role in tumor recurrence, chemoresistance and radioresistance [8–11]. However, knowledge regarding the role of candidate CSCs in radioresistance of HCC is limited. Regarding radioresistance associated with CSCs, a previous study reported that glioma stem cells promote radioresistance via preferential activation of the DNA damage response [12], and another study demonstrated that radioresistance is associated with reactive oxygen species (ROS) levels in CSCs [13]. We recently demonstrated that CD133-expressing liver cancer cells following radiation exposure showed higher activation of the MAPK/PI3K signaling pathway and reduced ROS levels compared with CD133 (−) liver cancer cells [14]. However, the mechanism by which irradiation maintains or reinforces the invasion and migration capabilities of CSCs, which reflects the metastatic potential of tumor cells, remains to be explored. A previous study demonstrated that radiation enhanced HCC cell invasiveness by MMP-9 expression through the PI3K/Akt/NF-kappaB signal transduction pathway [15]. Additionally, another study showed that radiation enhances the long-term metastatic potential of residual HCC through the TMPRSS4-induced epithelial-mesenchymal transition in nude mice [16]. However, whether activation of a particular gene related to liver CSCs can lead to metastasis in HCC remains unclear.

A disintegrin and metalloproteinase (ADAM), also known as TNF-α converting enzyme (TACE), plays a key developmental role by processing numerous growth factors and growth factor receptors [17, 18]. Studies have shown that ADAM17 is a potent sheddase of the epidermal growth factor (EGF) family of ligands and regulates EGFR activity in a variety of tumors [19, 20]. Additionally, ADAM17 plays important roles in tumor progression [21], hypoxia-induced tumor cell invasiveness [22] and hypoxia-induced cisplatin resistance [23]. In the present study, we found that ADAM17 was increased in irradiated liver CSCs, suggesting their involvement in the metastatic mechanism of HCC, and furthermore, this metastatic potential of liver CSCs may be decreased by ADAM17. Moreover, aberrant Notch signaling was reportedly related to tumorigenesis, self-renewal of CSCs and metastasis in various human tumors [24], and its downregulation was found to inhibit HCC cell invasion through inactivation of matrix metalloproteinase 2 (MMP-2), MMP-9 and vascular endothelial growth factor (VEGF) [25]. However, how ADAM17 regulates Notch signaling in liver CSCs after irradiation remains unclear.

In the present study, we explored whether ADAM17 in CD133-expressing liver CSCs plays a key role in radiation-induced tumor cell invasiveness or the metastatic potential of HCC.

## RESULTS

### The CD133-expressing Huh7 cell subpopulation exhibited metastatic potential with radioresistance properties

**Figure 1:**
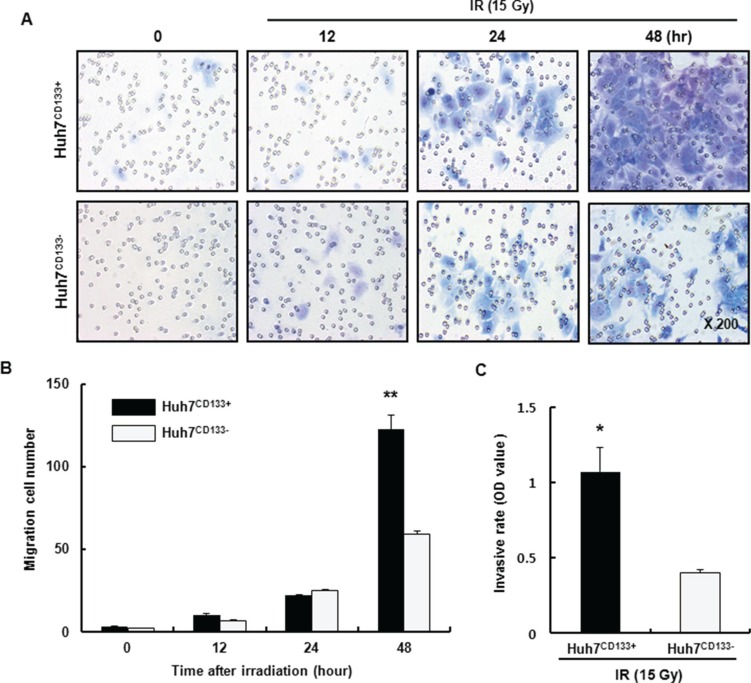
Migration and invasion assay of Huh7^CD133+^ and Huh7^CD133−^ cells after 15- Gy irradiation (**A**) The migratory capacity of Huh7^CD133+^ and Huh7^CD133−^ cells was assessed at 12, 24 and 48 h using a Transwell migration assay after 15- Gy irradiation. (**B**) The numbers of migrated Huh7^CD133+^ and Huh7^CD133−^ cells was compared. The results are expressed as means ± SD of three independent experiments. **P < 0.01, Huh7^CD133+^ (48 h) *vs*. Huh7^CD133−^ (48 h). (**C**) Invasive potential of Huh7^CD133+^ and Huh7^CD133−^ cells was measured using Matrigel-coated transwells after 48 h of 15- Gy irradiation. The results are expressed as means ± SD of three independent experiments. *P < 0.05, Huh7^CD133+^ *vs*. Huh7^CD133−^ cells.

Recent studies reported that irradiation enriches the population of cells expressing CSC markers [26]. In our previous study, we found that CD133 expression was significantly higher in 15- Gy irradiated Huh7^CD133+^ cells than in nonirradiated Huh7^CD133+^ cells. In addition, Huh7^CD133+^ cells may have greater anti-apoptotic activity due to increased Bcl-2 expression and radioresistance. These CSCs are radioresistant to both intrinsic and extrinsic determinants through various mechanisms, including preferential activation of the DNA damage response, lower cellular ROS levels and activation of survival signaling pathways [12]. Furthermore, in a growing tumor, CSCs regulate metastasis similar to normal stem cell processes [27]. The typical human HCC cell lines include Huh7, Hep3B, HepG2, Sk-hep1, PLC/PRF5 cell, among others. In this study, we isolated liver cancer stem cells (LCSCs) from various HCC cell lines using a PE-conjugated anti-CD133 antibody and a FACs system. In Supplementary Figure S1, we confirmed CD133-expressing LCSCs population in various HCC cell lines by FACs. The percentage of CD133 (+) LCSCs from the Sk-Hep1 cell line was only 0.1%, and therefore we could not use this cell line for further study. By contrast, the percentages of CD133 (+) LCSCs from Hep3B and PLC/PRF5 cell lines were 98.9% and 86.2%, respectively, and these cells also were inappropriate for further study. However, the ratios of CD133 (+) cells and CD133 (−) cells from the Huh7 cell line were 50.7% and 49.3%, respectively, which rendered it appropriate for use in further experiments. To determine whether Huh7^CD133+^ cells possessed greater metastatic ability than their Huh7^CD133−^ cell counterparts *in vitro*, we performed a migration assay of Huh7^CD133+^ and Huh7^CD133−^ cells sorted from the Huh7 cell line. At 12, 24 and 48 h after 15- Gy irradiation, the migrated cells in each well were stained using a Diff-Quik three-step stain kit (Figure 1A). Then, the numbers of migrated Huh7^CD133+^ cells and Huh7^CD133−^ cells were counted and compared (Figure 1B). As shown in Figure 1B, the migration ability of Huh7^CD133+^ cells was twofold higher than that of Huh7^CD133−^ cells at 48 h after 15- Gy irradiation. Next, to assess the invasion ability of Huh7^CD133+^ and Huh7^CD133−^ cells after 15- Gy irradiation, we measured and compared the optical density (OD) values obtained an invasion assay (Figure 1C). At 48 h after 15- Gy irradiation, the invasion ability of Huh7^CD133+^ cells was significantly higher than that of Huh7^CD133−^ cells. Taken together, these results suggest that irradiation exerts significantly greater stimulatory effects on the migration and invasion of Huh7^CD133+^cells than Huh7^CD133−^cells. Moreover, these results provided additional evidence that compared with Huh7^CD133−^cells, Huh7^CD133+^cell subpopulations are enriched in CSCs resistant to irradiation.

### Secretion of MMP-2 and MMP-9 by the CD133-expressing Huh7 cell subpopulation following irradiation

**Figure 2:**
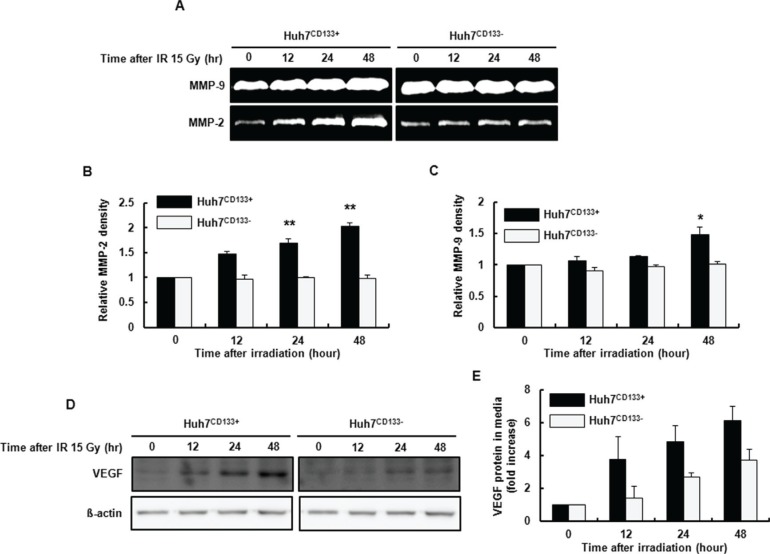
Gelatin zymography using conditioned Huh7^CD133+^ and Huh7^CD133−^ cell culture media after 15- Gy irradiation (**A**) MMP activities are visualized as white (clear) bands, corresponding to MMP-2 and MMP-9. (**B** and **C**) MMP-2 and MMP-9 band densities were quantified. The results are expressed as means ± SD of three independent experiments. **P < 0.01 *vs*. Huh7^CD133+^ and Huh7^CD133−^ controls. *P < 0.05 *vs*. Huh7^CD133+^ and Huh7^CD133−^ controls. (**D**) VEGF expression levels in Huh7^CD133+^ and Huh7^CD133−^ cells after 15- Gy irradiation were compared by Western blotting. (**E**) VEGF protein levels in conditioned media from Huh7^CD133+^ and Huh7^CD133−^ cells after 15- Gy irradiation were compared by ELISA.

MMP enzymes that degrade ECM proteins are a critical component of cell invasion in metastasis [15]. In this study, we investigated the gelatinolytic activities of MMP-2 and MMP-9 after 15- Gy irradiation. The radiation-induced MMP-2 and MMP-9 enzyme activities determined by gelatin zymography were significantly increased in Huh7^CD133+^ cell supernatants (Figure 2A–2C). As shown in Figure 2B, MMP-2 activity at 12, 24 and 48 h after 15- Gy irradiation was significantly increased in Huh7^CD133+^ cell supernatants compared with Huh7^CD133−^ cell supernatants. Additionally, MMP-9 activity at 24 and 48 h after 15- Gy irradiation was significantly increased in Huh7^CD133+^ cell supernatants compared with the control (Figure 2C). These results suggest that upregulation of MMP-2 and MMP-9 secretion after irradiation enhances the invasiveness of liver CSCs.

### Secretion of VEGF in CD133-expressing Huh7 cell subpopulations following irradiation

The expression of VEGF, an important growth factor stimulating angiogenesis, has been associated with tumor progression, metastasis and invasion in HCC [28] and other tumors [29]. A previous study reported that VEGF levels were enhanced by ionizing radiation in glioblastoma cells [30]. In this regard, we investigated whether the metastatic potential of liver CSCs after 15- Gy irradiation is associated with VEGF expression levels by Western blot and enzyme-linked immunosorbent assay (ELISA). As shown in Figure 2D, the rate of increase in VEGF expressions in irradiated Huh7^CD133+^ cells increased over time compared with irradiated Huh7^CD133−^ cells determined by Western blot. In addition, the increased VEGF levels in supernatants from irradiated Huh7^CD133+^ cells tended to increase over time compared with irradiated Huh7^CD133−^ cells albeit not significantly so (Figure 2E). These corresponding results from Western blots and ELISA indicate that CD133-expressing LCSCs after radiation have greater angiogenic potential, leading to enhanced metastatic ability.

### CD133 expression following irradiation increased metastatic potential *in vivo*

**Figure 3:**
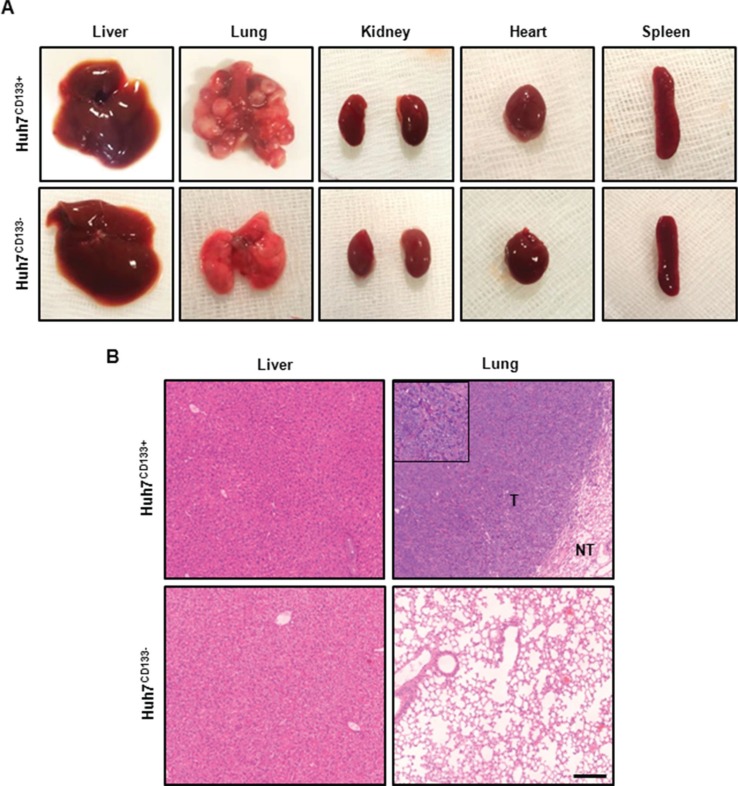
Gross photography of dissected organs and H & E staining from representative tumor from metastasis *in vivo* model (**A**) Representative images of dissected organs within the body after tail vein injection of irradiated Huh7^CD133+^ and Huh7^CD133−^ cells. Multiple nodules were observed in both lung but none in Huh7^CD133−^ injection group. In addition, no metastatic lesions were found in other organs except lung. (**B**) Representative H & E staining of the lung tissues in Huh7^CD133+^ injection group showed tumorous portion (T) and non-tumorous portion (NT). Tumors exhibited more aggressive invasion into surrounding lung tissue, undifferentiated and markedly pleomorphic tumor cells, suggesting poorly differentiated carcinoma. Scale bar, 200 μm.

To determine the effect of CD133-expressing LCSCs following radiation treatment on metastatic potential *in vivo* [31], irradiated Huh7^CD133+^ or Huh7^CD133−^ were injected via the tail vein. At 10 weeks after tumor cell injection via the tail vein, lung metastases were detected in 7 (70%) of the 10 mice injected with 1 × 10^6^ Huh7^CD133+^ cells after irradiation but not in the 10 mice injected with Huh7^CD133−^ cells (Table 1, Figure 3A). In the representative H & E staining of the lung tissues in Huh7^CD133+^ injection group, tumors exhibited more aggressive invasion into surrounding lung tissue, undifferentiated and markedly pleomorphic tumor cells, suggesting poorly differentiated carcinoma (Figure 3B). In addition, subcutaneous metastases were detected in 2 of 10 mice with 1 × 10^6^ Huh7^CD133+^ cells. However, no metastases were observed in the heart, kidney, spleen or liver in this group. These findings suggest that CD133-expressing LCSCs after irradiation have metastatic potential.

**Table 1:** Establishment of an *in vivo* metastasis model of human HCC cell line in nude mice

Cell line	Injection site	Cell number	Number of nude mice injected (n)	Weeks	Hematogenic metastases					Subcutaneous tumor formation
					Lung	Liver	Spleen	Kidney	Heart	
Huh7^CD133+^	i.v.	10^6^	10	10	7/10	0/10	0/10	0/10	0/10	2/10
Huh7^CD133−^	i.v.	10^6^	10	10	0/10	0/10	0/10	0/10	0/10	0/10

*i.v.: Intravenous injection.

### Differential profiling of metastasis-associated gene expression between Huh7^CD133+^ and Huh7^CD133−^ cells following irradiation

**Figure 4:**
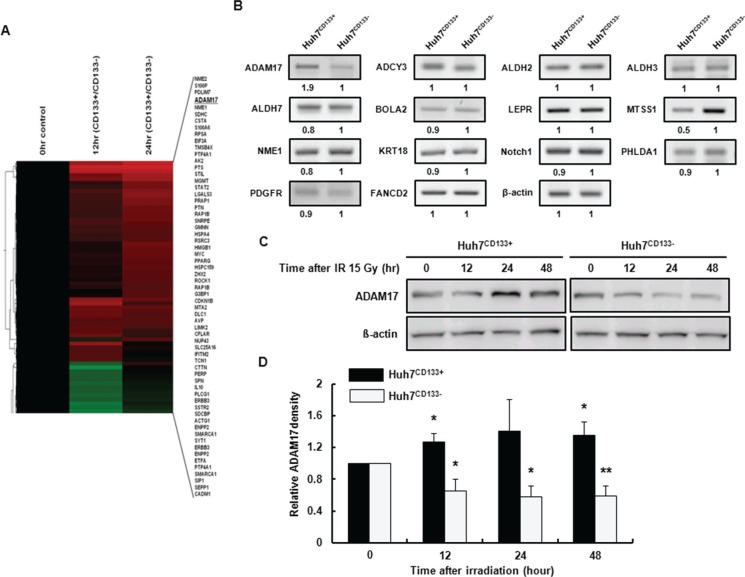
cDNA microarray analysis of the expression profiles of metastasis-associated genes in Huh7^CD133+^ and Huh7^CD133−^ cells at 12 and 24 h after 15- Gy irradiation (**A**) DEGs between Huh7^CD133+^ and Huh7^CD133−^ cells. The columns show DEGs in Huh7^CD133+^ and Huh7^CD133−^ cells at 12 and 24 h. Colors represent increases (red) and decreases (green) in CD133+ expression under each condition. (**B**) The expression levels of metastasis-associated genes were validated in Huh7^CD133+^ and Huh7^CD133−^ cells at 12 and 24 h after 15- Gy irradiation using RT-PCR. (**C**) Western blot analysis of ADAM17 protein levels after 15- Gy irradiation of Huh7^CD133+^ and Huh7^CD133−^ cells. β-actin was used as a loading control. (**D**) Band densities were quantified using the TINA imaging analysis software and normalized to β-actin expression. The data shown are the means ± SE of three independent experiments. **P < 0.01 *vs*. Huh7^CD133+^ and Huh7^CD133+^ controls. *P < 0.05 *vs*. Huh7^CD133−^ and Huh7^CD133−^ controls.

To evaluate how the cellular processes of Huh7^CD133+^ and Huh7^CD133+^ cells differ after irradiation, a cDNA microarray was used to analyze the gene expression profiles of Huh7^CD133+^ and Huh7^CD133−^ cells at 12 and 24 h after 15- Gy irradiation. The mRNA expression profiles of Huh7^CD133+^ and Huh7^CD133−^ cells were comparatively analyzed using a cDNA microarray containing 592 genes. Fifteen percent of the genes (89 genes) that displayed a greater than 1.5-fold change in Huh7^CD133+^ expression compared with the control Huh7^CD133−^ cells were associated with metastasis (Figure 4A). Grouping of the 89 metastasis-associated genes according to their biological function was performed between Huh7^CD133+^ and Huh7^CD133−^ cells at 12 and 24 h after irradiation. Among the genes increased in Huh7^CD133+^ cells 12 h post-irradiation, 13.5% (12 genes) were related to migration, 29.2% (26 genes) to cell adhesion, 25.8% (23 genes) to cell mobility and 7.8% to angiogenesis. Additionally, of the genes that were increased 24 h post-irradiation, 12.3% (11 genes) were associated with migration, 38.2% (34 genes) with cell adhesion, 32.6% (29 genes) with cell mobility, 9% (9 genes) with angiogenesis and 6.7% (6 genes) with VEGF signaling. Among the genes, for validation we selected 14 candidate reference genes (ADAM17, ADCY3, ALDH2, ALDH3, ALDH7, BOLA2, LEPR, MTSS1, NME1, KRT18, Notch1, PHLDA1, PDGFR and FANCD2) that showed overlapping upregulation at 12 and 24 h post-irradiation in Huh7^CD133+^ cells. Reverse transcription polymerase chain reaction (RT-PCR) for validation of selected candidate genes was performed in sorted Huh7^CD133+^ and Huh7^CD133−^ cells (Supplementary Table S1). However, only two genes showed at least a 1.5-fold difference: ADAM17 was increased 1.9-fold and metastasis suppressor 1 (MTSS1) was decreased 0.5-fold in Huh7^CD133+^ cells compared with Huh7^CD133−^ cells (Figure 4B). ADAM17 is a transmembrane and secreted protein with important roles in various metastatic functions, such as adhesion, migration, invasion and secretion of MMP-2 and MMP-9 [32]. In contrast, MTSS1 reportedly exerts its metastasis suppressor functions by acting as a scaffold protein that interacts with actin-associated proteins to regulate lamellipodia formation [33]. Therefore, we assessed the effect of ADAM17 on metastasis potential in CD133-expressing LCSCs after irradiation.

### Expression of ADAM17 in CD133-expressing liver CSCs following irradiation

To confirm the difference in ADAM17 protein levels in Huh7^CD133+^ and Huh7^CD133−^ cells after irradiation, cells were cultured and harvested at 12, 24 and 48 h after 15- Gy irradiation. Western blot was performed using antibodies against ADAM17. The ADAM17 protein level was increased significantly in Huh7^CD133+^ cells compared with the control at 12, 24 and 48 h after treatment with 15- Gy irradiation. However, the ADAM17 protein level was significantly decreased in Huh7^CD133−^ cells compared with the control (Figure 4C and 4D). Additionally, the MTSS1 protein level was not changed in Huh7^CD133+^ or Huh7^CD133−^ cells at 12, 24 and 48 h after irradiation, in agreement with the mRNA level based on RT-PCR, but no significant difference between the two cell groups was observed (Supplementary Figure S2A and S2B). These results suggest that overexpression of ADAM17 is associated with cell invasiveness, angiogenesis and migration, and could contribute to metastasis after radiotherapy for the treatment of HCC.

### ADAM17 mediates cell migration, invasion and wound healing in CD133-expressing liver CSCs following radiation exposure

**Figure 5:**
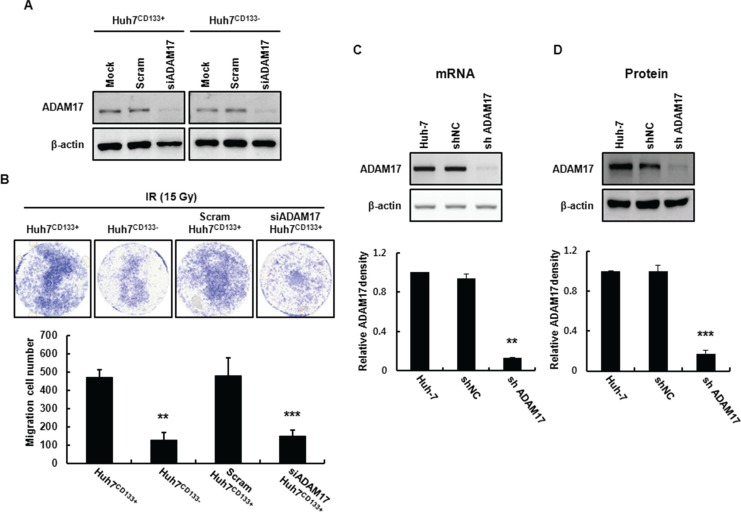
Establishment of an ADAM17-knockdown cell line using a lentiviral expression system (**A**) ADAM17 mRNA expression in Huh7^CD133+^ and Huh7^CD133−^ cells at 24 h determined by RT-PCR following transfection of ADAM17 siRNA. (**B**) Transwell migration assays of Huh7^CD133+^, Huh7^CD133−^, scram siRNA Huh7^CD133+^and ADAM17 siRNA Huh7^CD133+^ cells after 15- Gy irradiation. Images were captured at 48 h under × 40 magnification. The numbers of migrated Huh7^CD133+^, Huh7^CD133−^, scram siRNA Huh7^CD133+^and ADAM17 siRNA Huh7^CD133+^ cells were compared. **P < 0.01, Huh7^CD133+^ (48 h) *vs*. Huh7^CD133−^ cells (48 h). ***P < 0.001, Huh7^CD133+^ (48 h) *vs*. siADAM17 Huh7^CD133+^ cells (48 h) (**C**) ADAM17 mRNA levels in shNC and shADAM17 cells were analyzed using RT-PCR after infection with lentivirus. Band densities were quantified using the TINA imaging analysis software and normalized to β-actin expression. The data shown are the means ± SE of three independent experiments. **P < 0.01 *vs*. shNC cells and shADAM17 cells. (**D**) ADAM17 protein levels in shNC and shADAM17 cells were analyzed by Western blot analysis after infection with lentivirus. Band densities were quantified using the TINA imaging analysis software and normalized to β-actin expression. The data shown are the means ± SE of three independent experiments. ***P < 0.001 *vs*. shNC cells and shADAM17 cells.

**Figure 6:**
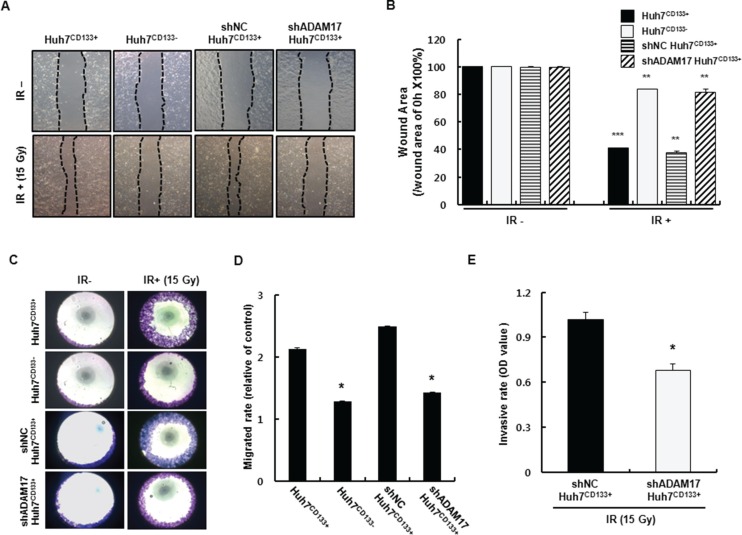
Changes in shADAM17^CD133+^ and shNC^CD133+^ cell migration after 15- Gy irradiation (**A**) Wound healing assay in Huh7^CD133+^, Huh7^CD133−^, shNC^CD133+^ and shADAM17^CD133+^ after 15- Gy irradiation. Four group of cells were plated on 24-well plates (1 × 10^5^ cells/well) and wounded with a disposable pipette tip. Wound closure was monitored for 48 h after irradiation. (**B**) Cell wound area percentages were analyzed using Optimas 6.5 software and compared with those at time zero. ***P < 0.001, Huh7^CD133+^ (48 h) *vs*. Huh7^CD133+^ cells (0 h). **P < 0.01, Huh7^CD133−^ (48 h) *vs*. Huh7^CD133−^ cells (0 h). **P < 0.01, shNC^CD133+^ (48 h) *vs*. shNC^CD133+^ cells (0 h). **P < 0.01, shADAM17^CD133+^ (48 h) *vs*. shADAM17^CD133+^ cells (0 h). (**C**) Migrated cells stained crystal violet (purple color). (**D**) Migrated cells (purple area) were quantified based on optical density (OD) values. *P < 0.05, Huh7^CD133+^ (48 h) *vs*. Huh7^CD133−^ cells (48 h). *P< 0.05, shADAM17^CD133+^ (48 h) *vs*. Huh7^CD133+^ cells (48 h). (**E**) Invasion assay of shADAM17^CD133+^ and shNC^CD133+^ cells was using Matrigel-coated Transwells. *P < 0.05, shADAM17^CD133+^ (48 h) *vs*. shNC^CD133+^ (48 h).

ADAM17 promotes tumor-induced angiogenesis through upregulation of growth factors such as VEGF via MAP kinase activation [34]. In the present study, ADAM17 in Huh7^CD133+^ cells (high metastatic potential) was knocked down using small interfering RNA (siRNA) to suppress metastasis. To assess whether the migration activity of CD133-expressing liver CSCs is influenced by ADAM17 silencing, Huh7^CD133+^ and Huh7^CD133−^ cells were transfected with 10 nM siADAM17 or 10 nM scrambled siRNA. ADAM17 expression after ADAM17 siRNA (10 nM) transfection was markedly suppressed (greater than 80%) in both cell types within 2 days, whereas no change in ADAM17 expression was observed in either cell type following treatment with scrambled siRNA (10 nM) (Figure 5A). Next, we investigated whether silencing of ADAM17 expression decreased the migration ability of liver CSCs after irradiation. In both cell types treated with mock siRNA, the migration ability of Huh7^CD133+^ cells was higher than that of Huh7^CD133−^ cells (Figure 5B), indicating the metastatic potential of CD133-expressing liver CSCs. Notably, we found that the migration ability of Huh7^CD133+^ cells was significantly reduced after silencing ADAM17 compared with mock control and scrambled siRNA, while that of Huh7^CD133−^ cells was unchanged (Figure 5B). These results suggest that ADAM17 expression in CD133-expressing liver CSCs plays a pivotal role in the metastasis of HCC after irradiation. To suppress ADAM17 expression effectively, stable cell lines expressing shRNA against human ADAM17 were generated by lentiviral transductions in Huh7 cells. As shown in Figure 5C and 5D, ADAM17 mRNA and protein levels were suppressed effectively in ADAM17 transfected Huh7 cells compared with cells expressing negative control shRNA (shNC). Furthermore, shADAM17^CD133+^ and shADAM17^CD133−^ cells following sorting using an anti-CD133 FACs antibody were suppressed compared with their shNC controls (Supplementary Figure S3A). In addition, CD133 protein levels were decreased in shADAM17^CD133−^ and shNC^CD133−^ cells after sorting (Supplementary Figure S3B). After successful establishment of the stable ADAM17 knockdown cell line, we investigated whether knockdown of ADAM17 suppresses the metastatic potential of CD133-expressing liver CSCs, such as their wound healing, migration and invasion, after 15- Gy irradiation. Four cell groups (Huh7^CD133+^, Huh7^CD133−^, shNC^CD133+^ and shADAM17^CD133+^) were examined in terms of their wound healing abilities analyzed after 15- Gy irradiation. The wound healing ability of shADAM17^CD133+^ cells was significantly inhibited at 48 h compared with shNC^CD133+^ cells and Huh7^CD133+^ cells. However, no changes in the wound healing ability of Huh7^CD133−^ cells were observed compared with the control (Figure 6A and 6B). Next, we performed migration assays. As shown in Figure 6C and 6D, silencing of ADAM17 by shRNA in Huh7^CD133+^ cells significantly inhibited migration ability at 48 h compared with that of shNC^CD133+^ cells and Huh7^CD133+^ cells. In addition, we performed invasion assays to determine the invasiveness of liver CSCs after irradiation. The invasion rate of shADAM17^CD133+^ was significantly lower than that of shNC^CD133+^ cells (Figure 6E). Moreover, we assayed VEGF levels in shADAM17^CD133+^ and shNC^CD133+^ cell culture media over time after irradiation. The VEGF levels in shADAM17^CD133+^ cell culture medium were significantly decreased at 24 and 48 h after irradiation compared with shNC^CD133+^ cell culture medium (Supplementary Figure S4). Taken together, these results demonstrated that silencing of ADAM17 significantly inhibited the migration and invasiveness of liver CSCs after irradiation, suggesting that suppression of ADAM17 after radiotherapy could prevent metastasis in HCC.

### Suppression of ADAM17 inhibits the migration ability of liver CSCs *in vivo*

**Figure 7:**
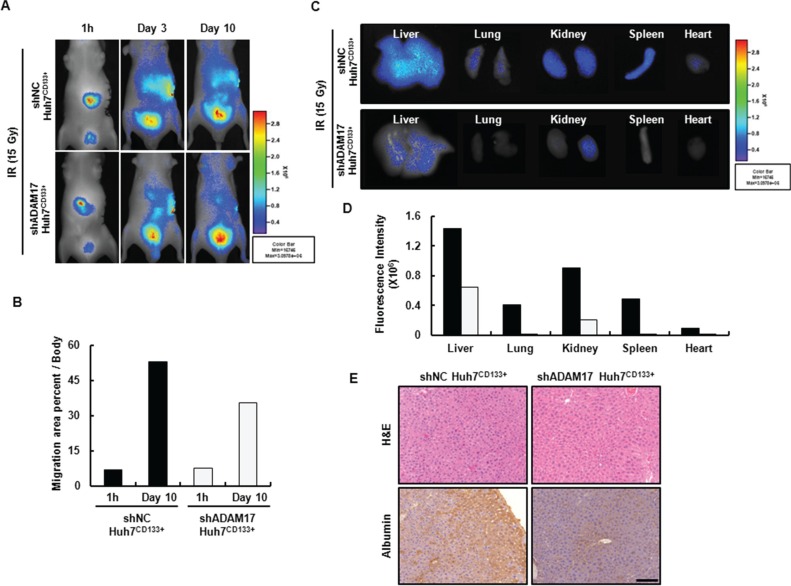
The real-time biodistribution of NIR-797-labeled shADAM17^CD133+^ and shNC^CD133+^ cells in nude mice (**A**) Fluorescent images of NIR-797-stained shNC^CD133+^ (up) and shADAM17^CD133+^ cell migration (down) 1 h and 3 and 10 days after intrasplenic vein injection in nude mice after 15- Gy irradiation (2 × 10^5^ cells/mouse). (**B**) Migration area percentages were analyzed using the Maestro Imaging System and Optimas 6.5 software. (**C**) Fluorescent images show the migration to other organs of NIR-797-stained shADAM17^CD133+^ and shNC^CD133+^ cells after 15- Gy irradiation following intrasplenic vein injection. (**D**) NIR signal intensity of mouse organs was obtained using the Maestro Imaging System. (**E**) Mice liver sections stained with H & E (TOP). Representative human albumin immunohistochemistry staining of cell migration in mice liver (Bottom). Scale bar, 100 μm.

Although *in vitro* studies elucidated the role of ADAM17 in the metastatic mechanism of CD133-expressing liver CSCs after irradiation, further *in vivo* evidence is necessary to confirm the role of ADAM17. First, we confirmed that the migration ability of irradiated Huh7^CD133+^ and Huh7^CD133−^ cells after intrasplenic vein delivery could be visualized by *in vivo* NIR fluorescence imaging (Supplementary Figure S5A). Cells were labeled with NIR 797 24 h before intrasplenic vein injection. Cells (2 × 10^5^) from each group were injected and the NIR signal intensity monitored. Ten days after injection, Huh7^CD133+^ and Huh7^CD133^ cells migrated out of the spleen toward other organs (liver and lung), and the cell migration area percentages were analyzed using the maestro imaging system (Supplementary Figure S5B). The migration area percentage of Huh7^CD133+^ cells increased to a greater degree than that of Huh7^CD133−^ cells after irradiation. Furthermore, the NIR fluorescent signal intensity of shNC^CD133+^ cells in the abdomen increased over time and spread throughout the body of nude mice, whereas the fluorescent signal intensity of shADAM17^CD133+^ cells slowly increased in the abdomen and their spread throughout the body was delayed (Figure 7A and 7B). These results demonstrated that the overall NIR fluorescent signal intensity was significantly reduced in the mice injected with ADAM17-suppressed Huh7^CD133+^ cells compared with ADAM17-nonsuppressed Huh7^CD133+^ cells. After imaging analysis, the mice were sacrificed on day 10 and the liver, kidney, spleen, lung and heart were harvested for imaging of the individual organs. NIR-stained shNC^CD133+^ cells migrated to the liver, kidney, spleen, lung and heart 10 days after intrasplenic vein injection, while NIR-stained shADAM17^CD133+^ cells slowly migrated to other organs after injection (Figure 7C and 7D). Furthermore, to identify the human-derived migrated cells in the liver of the nude mice after intrasplenic vein injection, immunohistochemical staining for human albumin was performed. The expression levels of human albumin were markedly higher in the livers of nude mice injected with shNC^CD133+^ cells compared with those injected with shADAM17^CD133+^ (Figure 7E). These results provide evidence that ADAM17 inhibits the migration and metastatic potential of CD133-expressing liver CSCs after radiotherapy and may serve as a prognostic marker of recurrence and metastasis of HCC following radiotherapy.

### The Notch signaling pathway is inhibited by suppression of ADAM17 in CD133-expressing liver CSCs

**Figure 8:**
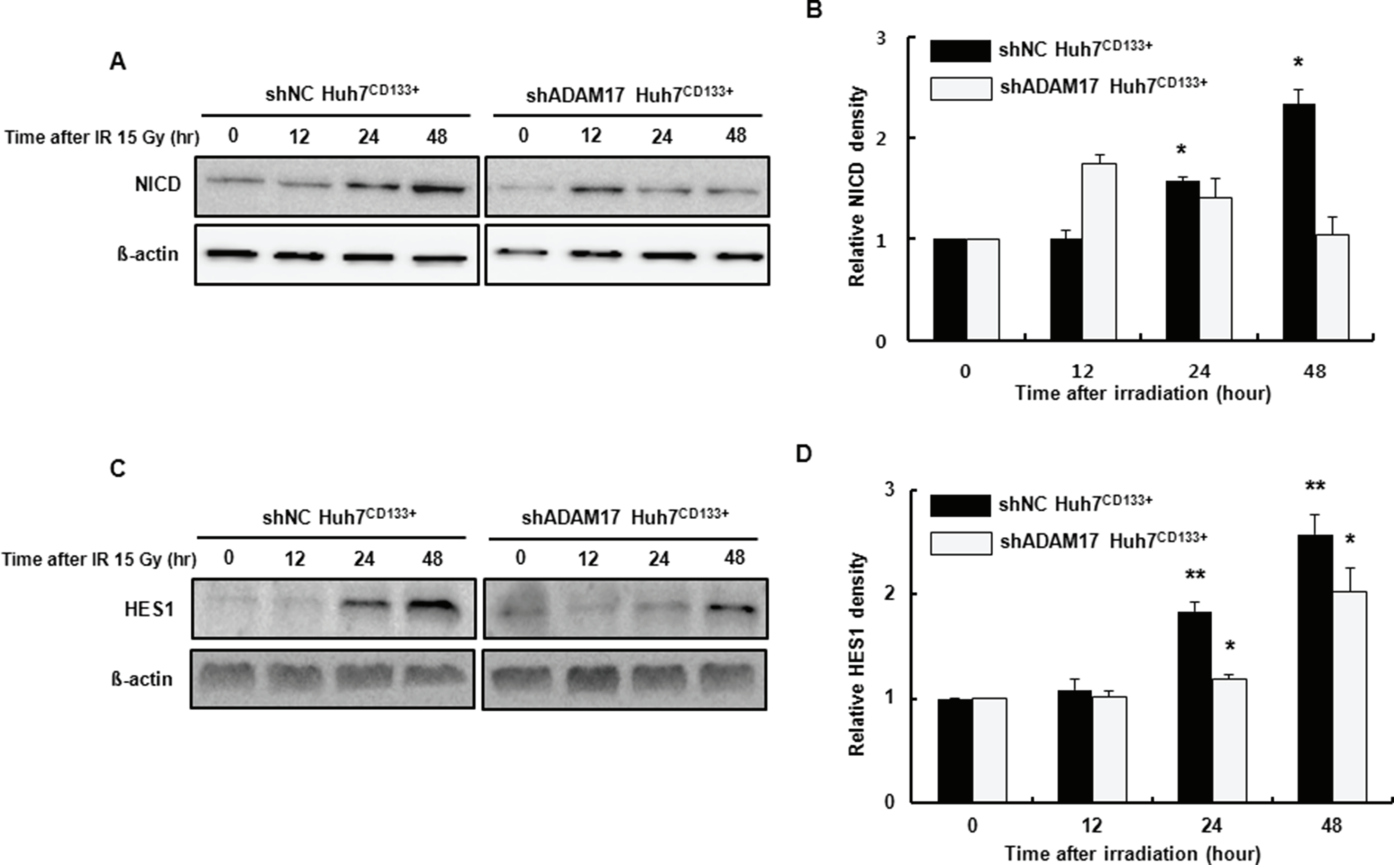
Notch signaling-associated protein expression in shADAM17^CD133+^ and shNC^CD133+^ cells following radiation exposure (**A**) Western blot analysis of NICD protein levels in shADAM17^CD133+^ and shNC^CD133+^ cells after 15- Gy irradiation. ß-actin was used as a loading control. (**B**) The band densities were quantified using the TINA imaging analysis software and normalized to β-actin expression. The data shown are the means ± SE of three independent experiments. *P < 0.05, shNC^CD133+^ *vs*. controls. (**C**) Western blot analysis of HES1 protein levels in shADAM17^CD133+^ and shNC^CD133+^ cells after 15- Gy irradiation. ß-actin was used as a loading control. (**D**) The band densities were quantified using the TINA imaging analysis software and normalized to β-actin expression. The data shown are the means ± SE of three independent experiments. *P < 0.05, shADAM17^CD133+^ *vs*. controls. **P < 0.01, shNC^CD133+^ *vs*. controls.

The Notch, Hedgehog and Wnt signaling pathways are involved in metastasis of liver CSCs to distant organs [35]. ADAM17 is an activator of the Notch pathway and is overexpressed in a variety of diseases, including cancers [36]. Among these pathways, Notch signaling reportedly affects angiogenesis [37] and metastasis [38]. The Notch intracellular domain (NICD) is cleaved from the plasma membrane and translocates into the nucleus forming a transcriptional activation complex after binding to CSL/RBPJk/Su(H) and Mastermind (MAML). This heteromeric complex activates the transcription of target genes such as HES [39]. In the present study, to determine the association of Notch signaling with liver CSCs with high metastatic potential after irradiation, we assessed the expression of NICD in the ADAM17-suppressed CD133-expressing liver CSCs after irradiation using Western blotting. NICD expression was reduced significantly in shADAM17^CD133+^ cells at 24 and 48 h after irradiation compared with that in shNC^CD133+^ cells (Figure 8A and 8B). Furthermore, to determine whether NICD translocates into the nucleus and activates transcription of genes, HES1 protein levels were determined using Western blotting. As shown in Figure 8C and 8D, HES1 expression was reduced significantly at 24 and 48 h after irradiation in shADAM17^CD133+^ cells compared with shNC^CD133+^ cells. Taken together, our results suggest that the Notch pathway is inhibited by suppression of ADAM17 during metastasis of CD133-expressing liver CSCs after radiotherapy.

## DISCUSSION

In the present study, we showed that stem-like cells play an important role in the radioresistance and metastatic potential of HCC. More specifically, the CD133-expressing Huh7 cell subpopulation showed greater resistance to sublethal irradiation and enhanced invasion and migration, which reflect metastatic potential. In the present study, our results demonstrated that irradiated CD133 (+) LCSCs have more predominantly metastatic potential than CD133 (−) LCSCs in the metastasis *in vivo* mice model which was established by tail vein injection of tumor cells. These results are consistent with a previous report that CD133-expressing cancer stem cells within gliomas are less sensitive to radiation-induced apoptosis and the fraction of CD133-expressing cells is increased in *in vitro* cultures after irradiation [12]. We recently revealed that CD133-expressing liver cancer cells have anti-apoptotic and radioresistance properties which are mediated by activation of the MAPK/PI3K signaling pathway [14]. Given the high tumorigenic capacity of HCC stem cells, disrupting the radioresistance of liver CSCs may enhance the efficacy of radiotherapy for HCC.

MMP activation in tumor metastasis allows tumor cells to access blood vessels, migrate to and invade target organs, ultimately leading to metastasis [40]. Among them, MMP-2 and MMP-9 play key roles in tumor invasion and metastasis because of their specificity for type IV collagen, which is the principal component of the basement membrane [41]. In our study, we demonstrated that radiation-induced MMP-2 and MMP-9 activities were more predominantly increased in Huh7^CD133+^ cell culture supernatant after irradiation. In addition, the secretion of VEGF, which plays an essential role in carcinogenesis and metastasis, was significantly increased. These results are consistent with a previous report that CD133-expressing glioma stem cells can promote tumor angiogenesis through hypoxia-induced elevated VEGF secretion [42, 43]. Taken together, our results showed that irradiation of an HCC cell line enriched in the CD133-expressing cell subpopulation led to metastatic potential, suggesting that radiotherapy rather than being curative may enhance tumor metastasis in HCC. Thus, the molecular mechanisms that regulate cell survival or death responses to ionizing radiation must be identified to develop novel therapies for HCC by targeting key signaling factors.

To investigate the mechanisms underlying the maintenance or reinforcement of the migration and invasion abilities of liver CSCs following irradiation, we performed comparative expression profiling of the Huh7^CD133+^ and Huh7^CD133−^ cell subpopulations. We identified 14 metastasis-associated genes responsible for cell invasion, migration or angiogenesis in enriched Huh7^CD133+^ cells after irradiation. Among them, ADAM17, which is involved in tumorigenesis, was significantly increased at both the mRNA and protein levels in enriched Huh7^CD133+^ cells following irradiation. A recent study showed that ADAM17 is decreased by hypoxia and contributes to hypoxia-induced cisplatin resistance *via* the EGFR/PI3K/Akt pathway in HCC [23]. In this study, we demonstrated for the first time that ADAM17 expression contributes to the invasion and migration of enriched Huh7^CD133+^ cells after irradiation. To further investigate the biological role of ADAM17 in the radiation-associated metastatic potential of CSC-enriched HCC cells, we suppressed ADAM17 expression using stable shRNA in enriched Huh7^CD133+^ cells after irradiation. Silencing of ADAM17 significantly inhibited the migration and invasiveness of enriched Huh7^CD133+^ cells after irradiation. Our results are consistent with a previous report that ADAM17 is associated with microvascular invasion in HCC, which results in disease progression [44]. Next, we determined whether these *in vitro* findings could be replicated *in vivo*. ADAM17-suppressed Huh7^CD133+^ cell migration was significantly inhibited compared with that of ADAM17-nonsuppressed Huh7^CD133+^ cells after irradiation based on the NIR fluorescence signals detected by *in vivo* real-time imaging analysis. These results suggest that ADAM17 is involved in the radiation-associated metastatic potential of CSC-enriched HCC cells after irradiation and thus may be a potential HCC therapeutic target. Moreover, a recent study reported that a specific anti-ADAM17 antibody showed anti-tumor effects in an ovarian cancer model *in vivo* [45]. In this regard, specific ADAM17 inhibition in combination with radiotherapy provides a promising therapeutic approach for patients with unrespectable HCC.

The Notch signaling pathway is involved in tumorigenesis and metastasis in various human tumors and plays an important role in CSCs [46]. Inhibition of Notch induces apoptosis and differentiation in CD133-expressing stem-like cells enriched from medulloblastoma cell lines [47]. Moreover, a recent report demonstrated that inhibition of the Notch signaling pathway inhibits HCC cell invasion by inactivating MMP-2, MMP-9 and VEGF [25]. A more recent study demonstrated that Notch inhibition in irradiated glioma stem cells dramatically increased cell death within 3 days after radiation, indicating a key role in post-radiation survival of glioma stem cells [48]. Another study examining the relationship between ADAM17 and Notch signaling showed that ADAM17 is a key enzyme for activation of the Notch signaling pathway, and that inhibition of its activity effectively promotes apoptosis and impairs invasion ability in renal cell carcinoma [49]. Based on the above results, we investigated the correlation between ADAM17 and Notch signaling in enriched CD133-expressing liver CSCs after radiation. In our study, Notch signaling was significantly reduced in irradiated CD133-expressing liver CSCs following stable knockdown of the ADAM17 gene. Additionally, HES1 expression was significantly reduced in shADAM17^CD133+^ cells after irradiation compared with shNC^CD133+^ cells. Collectively, our results suggest that inhibition of ADAM17 suppresses the Notch signaling pathway, resulting in decreased MMP-2, MMP-9 and VEGF, leading to inhibition of invasion or migration by CD133-expressing liver CSCs after irradiation. CD133-expressing CSCs were shown to be responsible for cell invasion and migration after radiation and their radiation-induced metastatic potential could be prevented by suppression of ADAM17 signaling, suggesting ADAM17 to be a potential target for radiotherapy in patients with HCC. In conclusion, our findings indicate that after irradiation of HCC cells CD133-expressing liver CSCs have significant metastatic capabilities, the maintenance of which might be inhibited by suppression of ADAM17. Therefore, suppression of ADAM17 may reduce the metastatic potential of liver CSCs via downregulation of ADAM17.

## MATERIALS AND METHODS

### Cell culture and irradiation

Human hepatoma cells (Huh7, Hep3B, SK-hep1 and PLC/PRF-5) were grown in Dulbecco's modified Eagle's medium (DMEM; Invitrogen, Carlsbad, CA, USA) supplemented with 10% fetal bovine serum (FBS, Invitrogen), 100 μg/mL penicillin and 0.25 μg/mL streptomycin (Invitrogen) and maintained in a humidified incubator at 37°C with 5% CO_2_. Cells were preincubated with serum-free DMEM and irradiated at 15- Gy using a cesium-137 source delivering 3.1 Gy/min (Gammacell 3000 Elan irradiator; Best Theratronics, Ottawa, Canada). After 15- Gy irradiation, the cells were plated at the same density for the *in vitro* study.

### RNA interference

A small interfering RNA (siRNA) method was used to knockdown ADAM17. The ADAM17 siRNA sense sequences were 5-CAUCAAGUACUGAACGUUUdTdT-3 and 5-AAACGUUCAGUACUUGAUGdTdT-3. Control cells were subjected to mock transfection with scrambled siRNA. Transfection was performed using G-fectin (Genolution, Korea) following the manufacturer's protocol.

### Flow cytometric analysis

Cells were incubated at 4°C with a phycoerythrin-conjugated anti-CD133/1 antibody (Miltenyi Biotec, Auburn, CA, USA) and analyzed by flow cytometry (FACSCalibur; BD Biosciences, San Jose, CA, USA). Huh7^CD133+^ and Huh7^CD133−^ cells were sorted using a FACSCalibur cell sorter. Isotype-matched mouse IgG was used as a control.

### DNA microarray-based gene expression profiling in CD133-expressing liver CSCs following radiation exposure

DNA microarray-based gene expression profiling in CD133-expressing liver cancer cells following radiation exposure was performed. The mRNA levels of Huh7^CD133+^ and Huh7^CD133−^ cells at 12 and 24 h post-irradiation and controls before irradiation were analyzed using the Illumina microarray platform (Sentrix Human HT-12v1 BeadChip; Illumina, San Diego, CA, USA). Total RNAs were extracted, reverse-transcribed, and amplified according to the manufacturer's instructions. The integrity of total RNA was analyzed using the 2100 Bioanalyzer (Agilent Technologies, Santa Clara, CA, USA). *In vitro* transcription was performed to generate cRNA, which was hybridized onto each array. The arrays were scanned using a Bead Station (Illumina). Under each condition, we performed gene expression profiling of two biological replicates obtained from independent cell cultures. The probe intensities were normalized using quantile normalization in beadarray 1. The probes were annotated using lumi 1.4, an R/Bioconductor package. The full dataset was submitted to Gene Expression Omnibus under submission number (GSE22247). Differentially expressed genes (DEGs) identified by cDNA microarray comparisons were assigned to associate the union of DEGs with GO Biological Processes (GOBPs) using the Database for Annotation, Visualization, and Integrated Discovery (DAVID). Several GOBPs and KEGG pathways in which the DEGs were significantly enriched (*p* ≤ 0.05) were identified, including metastasis-associated processes (motility/cytoskeleton, invasion, adhesion and angiogenesis).

### ADAM17 knockdown with stable short-hairpin siRNA (shRNA)

To establish a stable Huh7 cell line depleted of ADAM17 expression, cells were infected using a short-hairpin RNA (shRNA)-lentiviral infection system (Sigma-Aldrich Co.). We constructed an ADAM17 shRNA- carrying lentiviral vector, LV-ADAM17. The shRNA negative control-lentiviral particle (LV-NC) was used as a negative control. To generate stable cells, 1 × 10^5^ Huh7 cells were plated on 12-well plates, transduced with 5 MOI lentiviral particles (using 8 μg/mL hexadimethrine bromide [(Sigma-Aldrich Co.]), and incubated in DMEM containing puromycin (5 μg/mL) at 37°C with 5% CO_2_. Suppression of ADAM17 in stable cells was confirmed by RT-PCR and Western blot analyses.

### Western blotting

Protein extracts were resolved on 12% and 10% SDS–PAGE gels, transferred onto nitrocellulose membranes (Schleicher & Schuell, Dassel, Germany) and blocked in 5% skim milk. The following primary antibodies were used according to the manufacturer's instructions: anti-CD133 (Santa Cruz Biotechnology, Santa Cruz, CA, USA), ADAM17 (ab cam), NICD (Cell Signaling Technology, Beverly, MA, USA), MTSS1 (Santa Cruz Biotechnology), HES1 (Santa Cruz Biotechnology) and β-actin (Sigma-Aldrich Co.). After incubation with horseradish peroxidase (HRP)-conjugated anti-mouse or anti-rabbit secondary antibodies (Amersham Biosciences, Cardiff, UK), specific protein bands were visualized using enhanced chemiluminescence (Amersham Biosciences). The density of each band was measured using the TINA software.

### Migration assay

Huh7^CD133+^ and Huh7^CD133−^ cells were grown to 70% confluence in two 60-mm plates prior to experimentation for 12 h in 10% FBS after radiation treatment. Additionally, cells were washed and detached using 2 mM ethylenediaminetetraacetic acid (EDTA) in PBS. After complete detachment, cells were resuspended in serum-free medium. Migration of Huh7^CD133+^ and Huh7^CD133−^ cells was determined using a 24-well Transwell chamber system (Costar 3422, Corning Inc., NY, USA). Huh7^CD133+^ and Huh7^CD133+^ cells were seeded in the upper chamber at 3 × 10^4^ / mL in 0.2 mL serum-free DMEM medium. Medium supplemented with 10% fetal bovine serum was placed in the bottom well in a volume of 0.8 ml. After incubation for 12, 24 and 48 h, non-migrating cells on the upper side of the filter were removed with a cotton swab. The filters were stained with the Three-Step Stain Set (Diff-Quik; Sysmex, Kobe, Japan), and the cells that migrated to the lower side of the filter were counted under a slide scanner (Pannoramic MIDI; 3DHISTECH Ltd., Budapest, Hungary) in five randomly selected fields (× 200).

### Wound healing assay

Cell motility was assessed using a wound-healing assay and liver cell imaging. Huh7^CD133+^ and Huh7^CD133−^ cells were grown to a confluent monolayer on Nunclon 24-well plates (NUNC, MA, USA), serum-starved for 24 h and induced with dexamethasone for another 24 h. Then, monolayers were wounded using a disposable pipette tip. Wound closure was monitored for 48 h using the Cell-IQ real-time image capture system (Chip-Man Technologies Ltd, Tampere, Finland). To determine whether wound closure was the result of cell migration, cells were counted followed by Optimas 6.5 software system.

### Gelatin zymography

Huh7^CD133+^ and Huh7^CD133−^ cell culture media were mixed with non-denatured sample buffer (1 M Tris-Cl pH 6.8, 1% bromophenol blue, 20% SDS). The mixture was electrophoresed on a 10% polyacrylamide gel containing 1 mg/mL gelatin (Sigma-Aldrich Co., St Louis, MO, USA) and the gel was rinsed twice in distilled water with 2.5% Triton X-100 for 10 min and then incubated in activation buffer (50 mM Tris buffer, pH 7.4 containing 5 mM CaCl_2_ and 1 μM ZnCl_2_) at 4°C for 12 h. In gels stained with Coomassie brilliant blue R-250, MMP-2 appeared as a clear area and the band density was measured using the Multigage imaging software.

### Reverse transcription polymerase chain reaction (PCR)

Total RNA was prepared from shRNA LV-NC and ADMA17 shRNA lentiviral particles cells using TRIzol reagent (Invitrogen) according to the manufacturer's instructions. cDNA was synthesized from 2- μg total RNA and a random primer and Taq polymerase (Promega) were used for RT-PCR. The target primer sequences used were as follows: ADAM17 forward: 5′-TTT CAA GGT CGT GGT GGT GG-3′, ADAM17 reverse: 5′-TTC CCC TCT GCC CAT GTA TC-3′, and β-actin forward: 5′- GGC ACC ACA CCT TCT ACA ATG A-3′, β-actin reverse: 5′- CCC TCG TAG ATG GGC ACA GT -3′.

### Enzyme-linked immunosorbent assay (ELISA)

The expression levels of VEGF, were quantified in culture media using ELISA. In brief, culture medium was dispensed into 96-well microtiter plates (Nalgen Nunc) with coating buffer and incubated at 4°C overnight. The plates were then rinsed with PBS with 0.05% Tween-20 (PBST), reacted first with specific antibodies against VEGF at 37°C for 1 h and second with the supplied horseradish peroxidase (HRP)-conjugated goat anti-mouse IgG (Zymed, San Francisco, CA, USA; diluted 1:3,000). After washing three times with PBST, the plates were developed by incubation for 30 min at room temperature with 3,3′, 5,5-tetramethyl benzidine (TMB) as the substrate. The reaction was stopped by addition of 1 M H_2_SO_4_ and the absorbance at 450 nm was determined using a microplate reader.

### Histologic analysis

Formalin-fixed and paraffin-embedded liver specimens were sectioned and stained with hematoxylin and eosin (H & E). In addition, immunohistochemistry for human albumin was performed using standard protocols. After blocking endogenous peroxide activity with 1 wt% goat serum in PBS, the sections were incubated with primary antibody against human-albumin diluted 1:500 in antibody diluent at 4°C overnight. After washing, the peroxidase EnVision System (HPR rabbit/Mouse Envision System TM, DakoCytomation, Denmark) was applied at room temperature for 5–10 min. Peroxidase activity was detected using 3,3-diaminobenzidine tetrachloride and hematoxylin counterstaining.

### Animal and tumor cell inoculation

All animal experiments were performed in accordance with institutional guidelines and were approved by the Institutional Animal Care and Use Committee of The Catholic University of Korea. Four week-old Balb/c nude male mice (Central Lab. Animal Inc., Seoul, Korea) were housed in the animal facility for least 2 weeks before starting the experiments. To investigate the metastatic potential of irradiated LCSCs, a total of 10^6^ irradiated Huh7^CD133+^ or Huh7^CD133−^ cells per mouse were injected via the tail vein. Mice were assessed daily for tumor formation by palpation and survival was monitored daily. The mice were euthanized after anesthesia at 10 weeks to determine the extent of metastasis. The organs were separated and fixed in formaldehyde for H & E staining.

### *In vivo* imaging study

To assess the migration ability of liver CSCs *in vivo*, we performed a cell tracking *in vivo* real-time imaging study using near-infrared fluorescent magnetic NEO-LIVE^™^-Magnoxide 797 nanoparticles (NIR797; Biterials, Seoul, Korea) at 0.4 mg/mL. All procedures were performed according to the manufacturer's instructions. After irradiation, 2 × 10^5^ cells were marked with NIR-797 for near-infrared fluorescence imaging and then injected into the spleen of the nude mice, and fluorescence images were obtained using the Maestro Imaging System (Cambridge Research & Instrumentation Inc., MA, USA) for data acquisition and analysis.

### Statistical analysis

All data are expressed as the means ± standard error of the mean from at least three independent experiments. Significant differences between among test conditions were identified using the Student's *t*-test. *P < 0.05, **P < 0.01 and ***P < 0.001 was considered significant.

## SUPPLEMENTARY MATERIALS FIGURES AND TABLE


