# Supplementary material for: Role of ADAM17 in invasion and migration of CD133-expressing liver cancer stem cells after irradiation
Source: Oncotarget. 2016 Mar 16;7(17):23482–97. doi: 10.18632/oncotarget.8112 (PMC5029641; doi:10.18632/oncotarget.8112)
Supplement: Supplementary file 1 [file oncotarget-07-23482-s001.pdf]

Role of ADAM17 in invasion and migration of CD133-expressing liver cancer stem cells after irradiation

Supplementary Materials

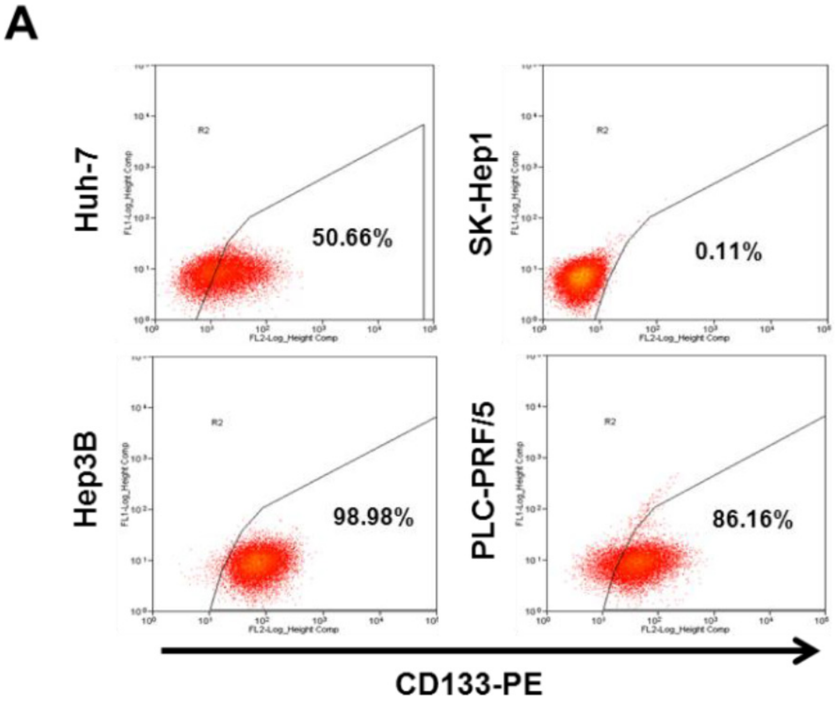

Supplementary Figure S1: Pattern of CD133 expression in four HCC cell lines. (**A**) The Huh-7, Sk-Hep1, Hep3B and PLC-PRF/5 HCC cell lines were labeled with PE-conjugated anti-CD133 antibody and sorted using flow cytometry.

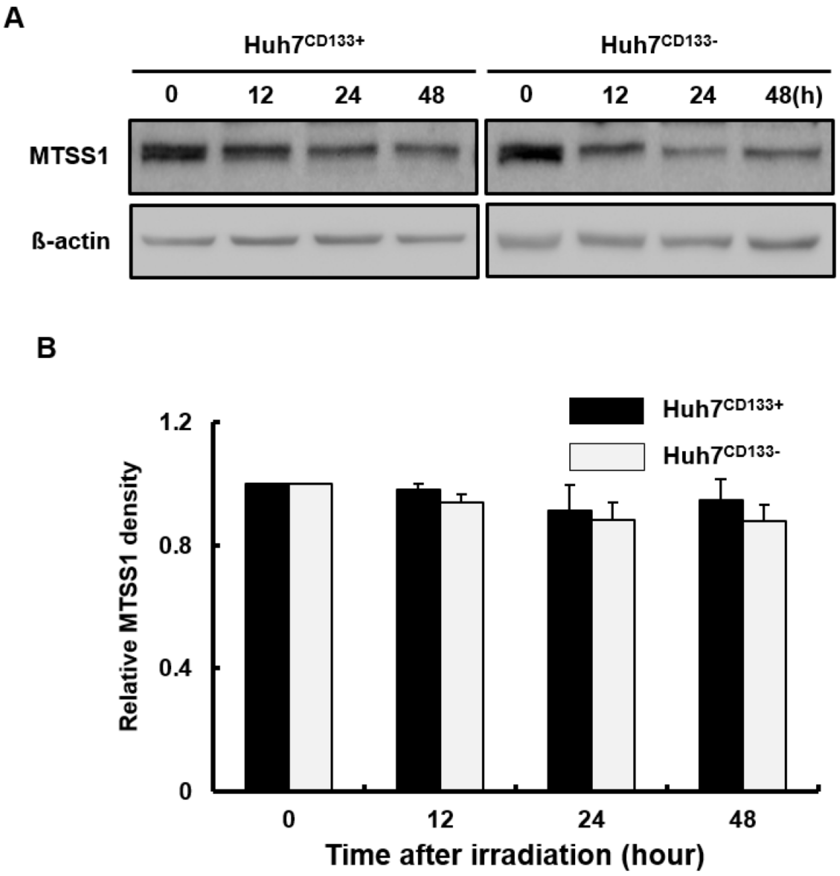

Supplementary Figure S2: MTSS1 protein levels were determined in Huh7^{CD133+} and Huh7^{CD133-} cells after 15- Gy irradiation. (**A**) Western blotting analysis of MTSS1 protein levels after 15- Gy irradiation of Huh7^{CD133+} and Huh7CD133- cells. β-actin was used as a loading control. (**B**) Band densities were quantified using the TINA imaging analysis software and normalized to β-actin expression.

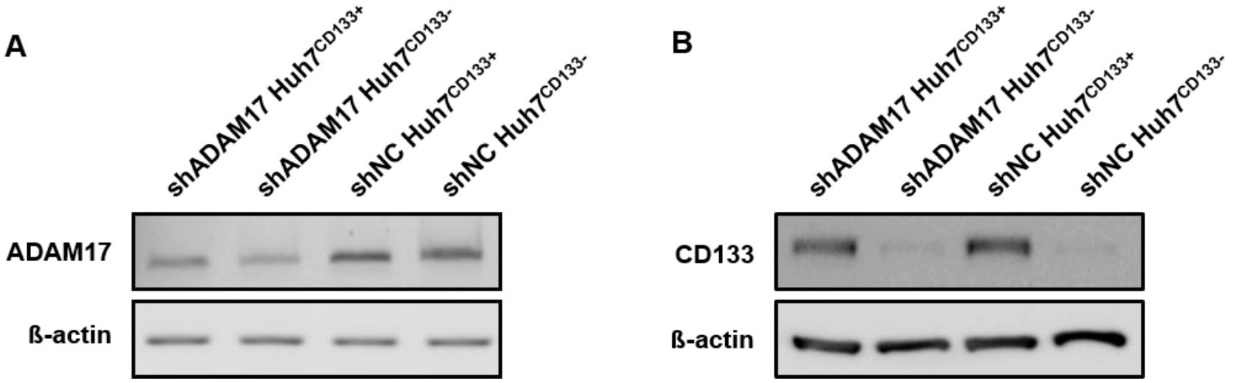

Supplementary Figure S3: Establishment of an ADAM17-knockdown cell line using a lentiviral expression system. (**A**) shNC and shADAM17 cells were sorted by FACS using a CD133/1 antibody. shNC^{CD133+}, shNC^{CD133-}, shADAM17^{CD133+} and shADAM17^{CD133-} cells were analyzed for ADAM17 mRNA after FACs. β-actin was used as a loading control. (**B**) CD133 protein levels in shNC^{CD133+}, shNC^{CD133-}, shADAM17^{CD133+} and shADAM17^{CD133-} cells were analyzed using western blot after FACs. β-actin was used as a loading control.

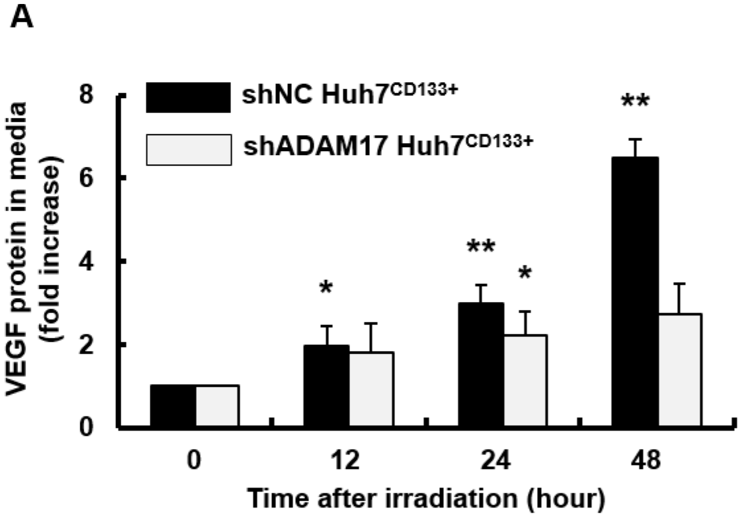

Supplementary Figure S4: Secretion of VEGF into the medium after irradiation. (**A**) VEGF protein levels were compared in shADAM17^{CD133+} and shNC^{CD133+} cell conditioned medium after 15- Gy irradiation using ELISA. The data shown are the means ± SE of three independent experiments. ***P* < 0.01 vs. shNC^{CD133+} and shADAM17^{CD133+} controls. **P* < 0.05 vs. shNC^{CD133+} and shADAM17^{CD133+} controls.

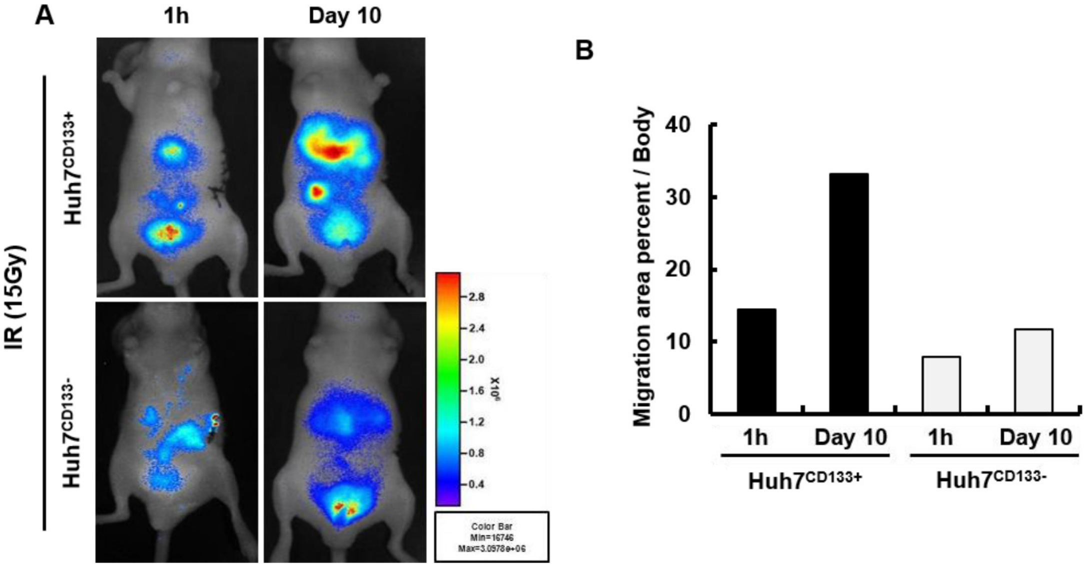

Supplementary Figure S5: The real-time biodistribution of NIR-797-labeled Huh7^{CD133+} and Huh7^{CD133-} cells in nude mice. (**A**) Fluorescent images of NIR-797-stained Huh7^{CD133+}cells (up) and Huh7^{CD133-} cells (down) migration after 1h and 10 days of intrasplenic vein injection in nude mice after 15- Gy irradiation (2 × 10^5 cells/mouse). (**B**) Migration area percent were analyzed using the Maestro Imaging System and Optimas 6.5 software.

Supplementary Table S1: Primer for RT-PCR

Gene	Forward primer sequence	Reverse primer sequence
LEPR	5′-AGT AAC GGT TCC ACA TCA ACT TG-3′	5′-GGC CTC AAA ATG TAA GAT GCT TAT AC-3′
MTSS1	5′-TCAAGAACAGATGGAAGAATGG-3′	5′-TGCGGTAGCGGTAATG-3′
ADAM17	5′-TTT CAA GGT CGT GGT GGT GG-3′	5′-TTC CCC TCT GCC CAT GTA TC-3′
PDGFRα	5′-GCT GTT TCT GTT GAC TTT TGA C-3′	5′-AAA CAA GGA ACT CAG AGA GGA-3′
PHLDA1	5′-CCA CAT CCA CAT CCA CAC TCT-3′	5′-AGG TGC TGC GGA GAA GCC GGT-3′
BOLA2	5′-CGC CTC GAG ATG GCA AGC GCG AAA-3′	5′-GGG AAT TCT TTC TGT CGC TCA CGT G-3′
ADCY3	5′-TTG ACT GCT ACG TGG TGG TCA TGT-3′	5′-TGC AGA GCA CGA AGA GGA TGA TGT-3′
ALDH2	5′-ACC ATC CCC ATT GAC GG-3′	5′-GAT CAG GTT GGC CAC AT-3′
ALDH3	5′-GGG AAA TTC ATG AAC AGT GGC-3′	5′-CAC GTC CGT GAG GAT GGT G-3′
ALDH7	5′-TCA AGC GGG TGC TGA CCC AG-3′	5′-CTC AGA GCA GTG TGC AGC-3′
KRT18	5′-AGT CTG TGG AGA ACG ACA TCC-3′	5′-TGG TGC TCT CCT CAA TCT GC-3′
NME1	5′-TTA ATC AGA TGG TCG GGG AT-3′	5′-GAT CTA TGA ATG ACA GGA GG-3′
FANCD2	5′-CCG GAA TAT TGG ATT CTC ACA T-3′	5′-GAA CTT TCA CTC CTG GTC CAT C-3′
Notch1	5′-GCC GCC TTT GTG CTT CTG TTC-3′	5′-CCG GTG GTC TGT CTG GTC GTC-3′
β-actin	5′-GGCACCACACCTTCTACAATGA-3′	5′-CCCTCGTAGATGGGCACAGT-3′

F, forward primer; R, reverse primer; RT, reverse-transcription primer.
